# Analytical and Experimental Study on Cold-Formed Steel Built-Up Sections for Bending

**DOI:** 10.3390/ma15207140

**Published:** 2022-10-13

**Authors:** R. Sujitha, N. Sunmathi, R. K. Manikandan, J. Arunprasad, S. Rajkumar, Shubham Sharma, Kamal Sharma, Changhe Li, Elsayed Mohamed Tag Eldin

**Affiliations:** 1 Department of Civil Engineering, Dhanalakshmi Srinivasan Engineering College, Perambalur 621212, India; 2 Department of Mechanical Engineering, Dhanalakshmi Srinivasan Engineering College, Perambalur 621212, India; 3 Department of Mechanical Engineering, Faculty of Manufacturing, Institute of Technology, Hawassa University, Hawassa P.O. Box 5, Ethiopia; 4 School of Mechanical and Automotive Engineering, Qingdao University of Technology, Qingdao 266520, China; 5 Mechanical Engineering Department, University Centre for Research and Development, Chandigarh University, Mohali 140413, India; 6 Institute of Engineering and Technology, GLA University, Mathura 281406, India; 7 Faculty of Engineering and Technology, Future University in Egypt, New Cairo 11835, Egypt

**Keywords:** cold-formed steel, ANSYS software, bending test, complex edge stiffener

## Abstract

In the construction of steel structures, the two most common types of structural members are hot-formed and cold-formed members. This paper mainly describes the analytical and experimental research on the strength and characteristics of CFS bolted built-up sigma sections having different structural arrangements under bending. The cross-sectional dimensions for the parametric study were selected by the sizes available in the market. In this paper, ANSYS workbench software was used to perform FE modeling and observe the local, flexural, and interaction of these buckling. Then, experimental study was performed by varying the arrangement of open section beams between face-to-face and back-to-back, connected using bolts or fasteners different spacings. Further, we conducted bending tests on cold-formed steel built-up members having simple edge stiffeners in the middle. Comparing both analytical and experimental studies, the results indicate that the back-to-back connected built-up beam section provides a flexural capacity higher than the face-to-face built-up section. Moreover, increasing the bolt spacing enhanced the load-carrying capacity of back-to-back sigma section built-up beams. It has also been discovered that the flexural strength of beams is primarily determined by bolt spacing or itsposition.

## 1. Introduction

Over the last few decades, cold-formed steel sections with thin walls have been enhanced in civil constructions for lighter loads. Thanks to their lightweight characteristics, a high strength/weight ratio gives greater stiffness, recyclability, uniform and smooth finish, and provides an aesthetic appearance and ease of fabrication. They also reduce the heavyweight of steel structures while having the strength and designer’s requirements met. CFS built-up cross-sectional members can be made economically stronger structures and more efficient to increase their load-carrying capacity. Generally, CFS with open section and thin thickness is largely subjected to deforming and buckling. Under bending, cold-formed steel can experience diverse form of instabilities like web buckling, local, flexural, distortional, and lateral-torsional buckling and interaction. Local and distortional buckling are the most common mechanisms of beam failure. These failures can be eliminated or deferred by proposing a new section with different shapes and cross-sections andthe formation of two or more open sections connected by bolts. Thus, a new design notion is familiarized by enumerating the stiffened element at the flange/web and simple edge stiffeners at the intermediate flanges to causea substantial change in flexural and other behavior beams.CFS forms in open-section are channel, sigma, and C and Z sections, among others, with varying thicknesses from 0.5mm to 3.0mm. Bolts are designed according to the following codes for cold-formed steel design: EN1993-1-3, EN1993-1-8, and AISI S100 (201). The investigation shows the simulated two-run flexural test on CFS of C and Z sections beams of unbraced members using the finite element (FE) model. Local and distortional buckling of specified beams is avoided through the fastened panel and specific fastener. The corrugated panel fixed at the compression flange was removed at a singlemoment. In the end, the specimens failed under distortional buckling [1]. An experimental comparison was carried out on a CFS bolted beam with two different channel sections, that is, outstand stiffener and extended stiffener, by injecting bolts and nuts on stiffener and web element, respectively. They determined the deformation of load and flexural strength of the beam specimens and concluded that the CFS beam with an extended stiffener has the best flexural strength [2]. The study on bending shows the aftermath collapse behavior of the U section and Ω section of thickness of 0.6mm and 1.5mm, respectively, and steel grades S275 and S375, respectively.

The test result suggeststhe plastic hinges and moment rotation m-ⱷ diagram have high deformation [3]. Additionally, the FEM in ANSYS was used in the numerical simulation analysis. The simulation propagates the forming of hinges under pure bending. It creates moment rotation curves of beams and thorough details about the stress and strain of beams during the entire loading process. A comparative study on experimental and analytical study observed that the FEM analysis of U-section has a deformed shape, coinciding with the plastic hinge obtained experimentally. Moreover, the numerical simulation shows that the maximum moment reacheda higher value than the experimental test [4]. This study works on a relative survey of different types of intermediate stiffeners. These critical stresses are analysed by finite strip software CUFSM and the ultimate resistance of CFS is analysedby the direct strength method. Among several shapes, such as rectangular, V, and arc shapes, it is observed that a V-shape stiffener on the web with an angle of 100° is optimum, and it has been found that an increase in height increases the ultimate resistance of the moment [5]. This paper explained that the increase in bending moment of amalgamation of two vertical and two horizontal elements back-to-back of CFS built-up beams increases with the yielding strength and thickness of the steel. Moreover, the stiffener with a reinforcing web is directly proportional to moment carrying capacity and inversely proportional to local web buckling [6]. This deals with the simple lipped channel of a stiffened and unstiffened element. The flexural strength and aspect of the beam increase in the stiffened element at the flange/web compared with in the unstiffened element because of higher hindrance against torsional buckling and greater hindrance in a moment of inertia [7]. The CFS beam built-up I section of the lipped channel with an intermediate and edge web stiffener leads to higher strength because of the considerably reduced LB and TB compared with the I beam without a web stiffener [8]. Comparing the open and closed C-channel of drop flanges of a CFS built-up beam, it ends with a gradual crash, and further investigation needs to be carried out for thin walls [9]. The author detailed the numerical study ABACUS developed for FE modelling and experimentally tested related to lateral-torsional buckling. They have developed a novel design equation to forecast moment capacities precisely. They also suggested a design curve named buckling curve ‘a’ of a CFS beam [10,11,12]. The study demonstrates that there have been few studies on cold-formed steel under bending, and there have been even less studies on lipped channels. Hence, the scope of this study is to inspect the behavior of the CFS built-up sigma section stiffened at the intermediate and edges in bending. On the whole, three sigma sections are taken. They are connected symmetrically using bolts of the varied spacing by labeling with names B2B100, where B2B refers to back-to-back, and 100 refers to the bolt spacings in mm, as well as face-to-face F2F100 and B2B157, respectively. Analytical studies are carried out using finite element modeling software ANSYS workbench 16.1. The developed analytical results tend to verify the experimental results. These results are useful to conclude on the mechanical strength properties and other behavior of CFS built-up beams.

## 2. Material and Methods

### 2.1. Analytical Investigation

A finite element modeling of ANSYS WORKBENCH16.1 software was used for the numerical study. The finite element was used to convert the physical structure into a complex system having point group termed nodes, making a grid called a mesh. The structure’s ability to react to certain loading conditions will be computerized by the mesh, which contains the material and structural element properties. SHELL181 was preferred to simulate the buckling behavior as the section thickness is less than one-tenth of the elemental dimension.

### 2.2. Selection of Section

The sectional properties of the selected sections were obtained from CUFSM software 4.5, developed by Schafer, Johns Hopkins University (Baltimore, MD, USA). As the investigation aims to obtain the best profile, a shorter span of 1200 mm and a sectional area of 840 sq. mm were chosen for all beams. The C/S dimensions were fixed, based on the AISI specification for cold-formed steel constructions, and covered the feasible range of sigma section beams already utilized in the industry to minimize local buckling. Even though the AISI specification for cold-formed steel structures (AISI: S100-2007) does not set limits for D/b ratios, Kankanamgeand Mahendran (2012) [13] provided guidelines for selecting D/b ratios of cold-formed steel beams in the range of 2 to 3.3.

### 2.3. Connection Requirements

The basic fabricated sigma sections are connected using bolt connections in different B2B and F2F methods. The bolts were designed according to the following codes for cold-formed steel design: EN1993-1-3, EN1993-1-8, and AISI S100 (2012). The details of the bolts and specifications used are as follows: diameter of 8 mm, with MS 4.6 grade, yield strength of 225 Mpa, and tensile strength of 400Mpa. Other connection requirements include edge distance, bolt spacing, bolt hole diameter, shear strength, and several bolts based on the code mentioned above.

### 2.4. Finite Element Modeling Boundary and Loading Conditions

In finite element modeling, selecting the mesh density is very important, as an increase in mesh density increases the convergence of the obtained results. Various mesh studies were taken out for the enhanced built-up section and it was found that a suitable mesh size for the whole section is 5 mm × 5 mm. When creating the model of the beam specimens, the C/S of the beam is generated in the X-Y plane and the span of the beams is on the Z-axis. The ends of the beam are supported. Hence, on one end, translations in Ux, Uy, and Uz are constrained. In the experimental setup, the bearing plate with a width of 50mm is used at the point of support and loading. Hence, in the FEA model, the boundary conditions are given to all of the nodes within a 50mm width on either end of the beam, and the loading is given in terms of displacement on the top compression flange at the points of loading for a width of 50mm.Finite element analysis transforms the physical structure into a complicated system of nodes that form a grid known as a mesh. This mesh is designed to include the material and structural attributes that dictate how the structure will respond to different loading circumstances. It can perform both linear and nonlinear analyses. Linear models have basic parameters and are based on the assumption that the material is not plastically deformed. Non-linear models incorporate material straining beyond its elastic limits.

**Figure 1:**
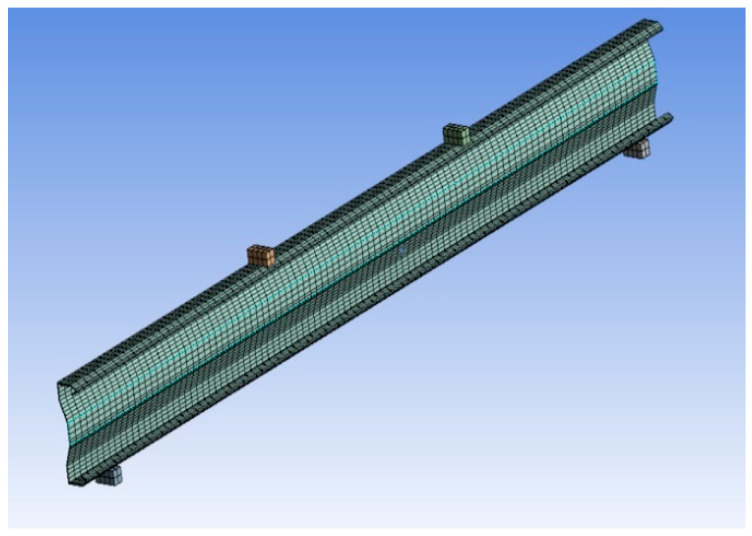
Meshing.

**Figure 2:**
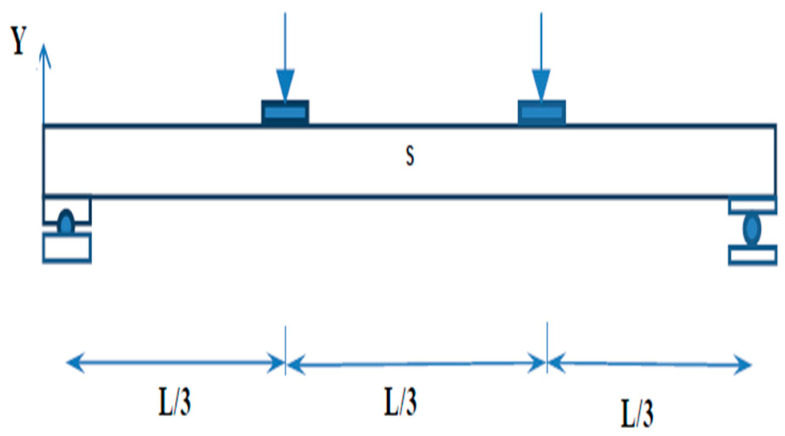
Physical model of the test setup.

The FEA process begins with the production of a geometric model of the structure, which is then subdivided into smaller forms that are joined at certain nodal points. Stress–strain relationships are more easily approximated in this manner. Finally, each element’s material behaviour and boundary conditions are included, and the analysis is run. Given the costs and time required for the production and testing of physical models, FEA provides a cost-effective solution to many engineering challenges (Figure 1 and Figure 2).

### 2.5. Results of Analytical Study

The six CFS built-up beam sections are checked under FEM, the loading is applied as displacement, and the amount of load used is taken as total force reaction at the displacement location. The parameters considered for the present study are (1) buckling mode of the section, (2) stress distribution of the section, (3) ultimate load carrying capacity, and (4) load–deflection behavior.

### 2.6. Buckling Mode and Stress Distribution of the Specimen

The buckling shape and stress distribution for all three specimens are shown in the following figures: (a) modeling of the specimen, (b) buckling mode of the specimen, and (c) stress distribution of the section.

#### 2.6.1. Specimen B2B100

**Figure 3:**
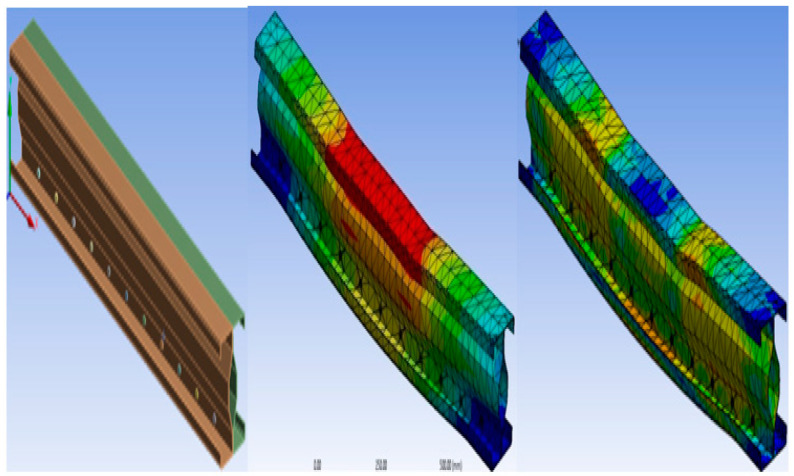
Buckling and stress distribution of B2B100.

Figure 3 shows the Buckling and stress distribution of B2B100 Specimen.

#### 2.6.2. Specimen F2F100

**Figure 4:**
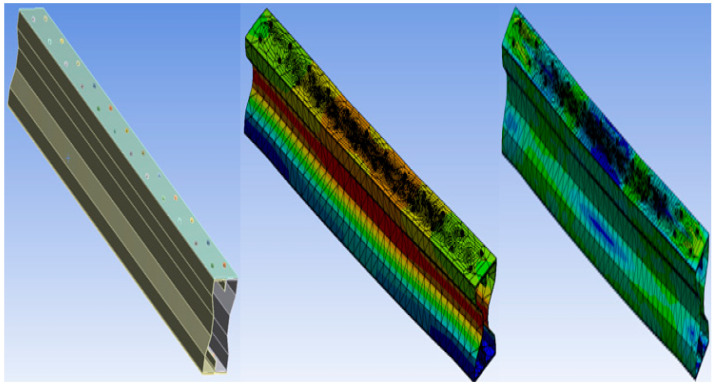
Buckling and stress distribution of F2F100.

Figure 4 shows the Buckling and stress distribution of F2F100 Specimen.

#### 2.6.3. Specimen B2B157

**Figure 5:**
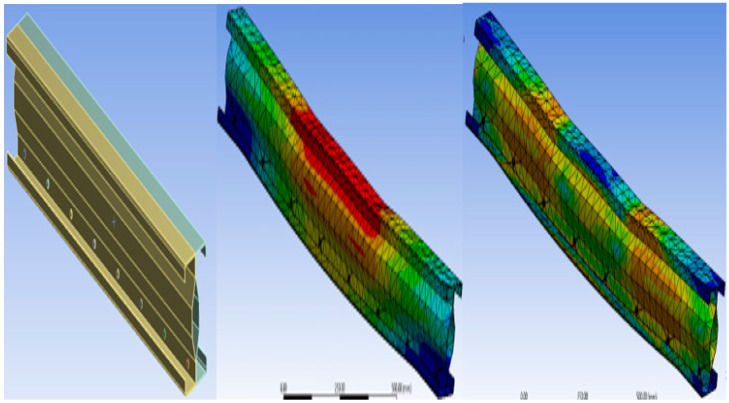
Buckling and stress distribution of B2B157.

Figure 5 shows the Buckling and stress distribution of B2B157 Specimen.

### 2.7. Ultimate Load Carrying Capacity

The three specimens were modelled and analysed using the ANSYS 16.1. From the analytical result, the ultimate load-carrying capacity of each specimen was obtained. The ultimate load of the built-up sections is presented in Table 1.

**Figure 6:**
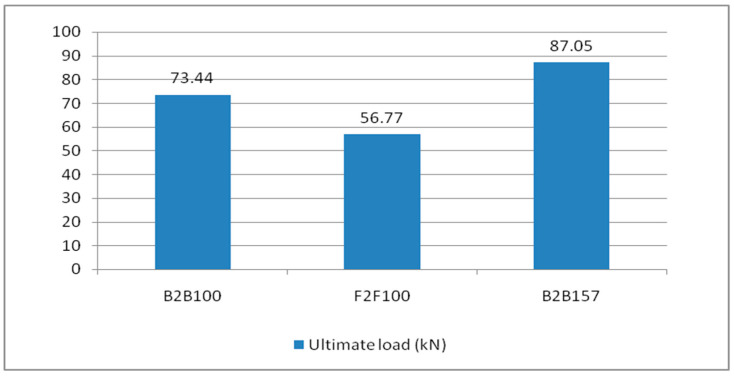
The ultimate load capacity of the built-up section.

From the table, it is found that the B2B100 standard section has a critical load of 70 kN. The most effective section is B2B157, having a critical load of 85 kN, which is a 21.4% increase in a load-carrying capacity relative to the standard section B2B100. The failure modes of the suggested CFS sections are determined to be local buckling and lateral-torsional buckling. The specimens’ maximum load-carrying capacity is compared and shown in the Figure 6.

### 2.8. Load-Deflection Behavior

**Figure 7:**
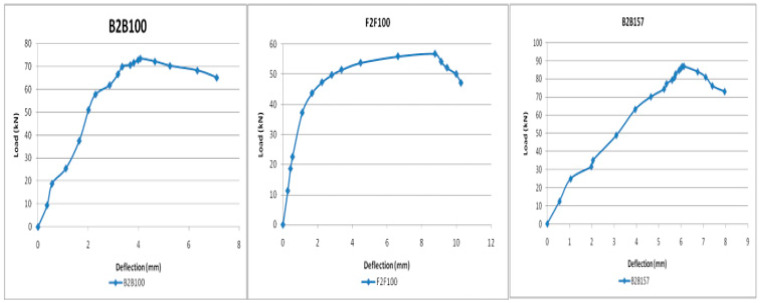
Load vs. deflection behavior.

In this study, load versus deflection curves were plotted to study the critical flexural strength of the beams. The load and deflection values obtained from the Ansys workbench 16.1 are presented as follows: the B2B100 section has an ultimate load of 73.44 kN and the F2F100 section has a maximum load of 56.77 kN, which is a 22.6% decrease in the load-carrying capacity compared with the standard section B2B100 and, finally, B2B157 has an ultimate load of 87.05 kN which is a 15.6% increase in the load-carrying capacity compared with the standard section B2B100. The load versus deflection behavior is presented (Figure 7).

## 3. Experimental Setup

**Figure 8:**
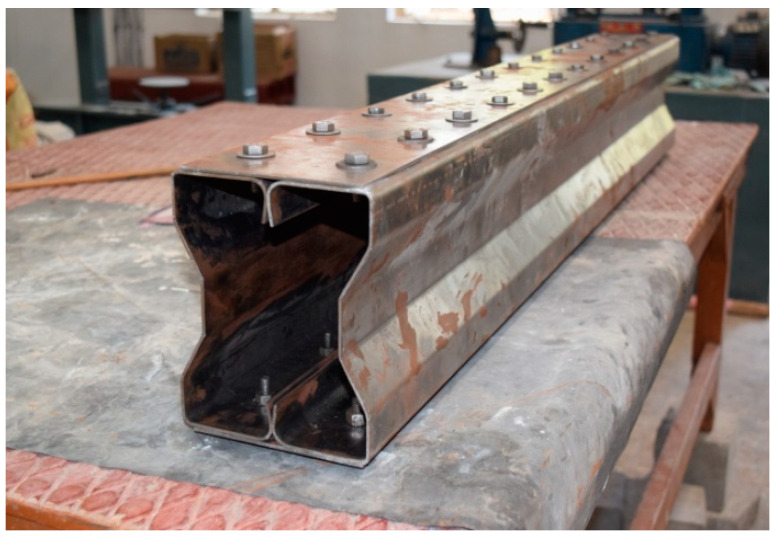
CFS beam specimens before testing cold-formed built-up sections.

The beam specimens are placed in a loading frame of 500 kN in two-point loading for testing. One end of the support is hinged, while the other end is a roller. The specimens are similarly regularised to apply the load vertically. The beam is laterally constrained at the support to prevent lateral movement. The overall stability of the experimental equipment is verified, and a small amount of preload is applied to seat the specimen in position before it is released. All of the instruments are in place and the load is applied using a hydraulic jack with a capacity of 200kN. All relevant data are recorded, including the applied force and the specimen’s deformation. All of the tests are completed to the point of failure. Dial gauges of least a count of 0.01 mm with a maximum travel of 50 mm were kept to measure deflection at the mid-point. A proving ring of 100kNwas used to measure the load applied in the section (Figure 8).

### 3.1. Experimental Results

The experimental investigation was carried out for specimens B2B100, F2F100, and B2B157. Loading was applied as shown in the figure and the amount of load applied was obtained from the proving ring. The deflection value was taken from the dial gauge. The parameters considered for the present study are (a) buckling mode of the section, (b) ultimate load carrying capacity, and (c) load–deflection behavior.

### 3.2. Buckling Modes of the Specimen

**Figure 9:**
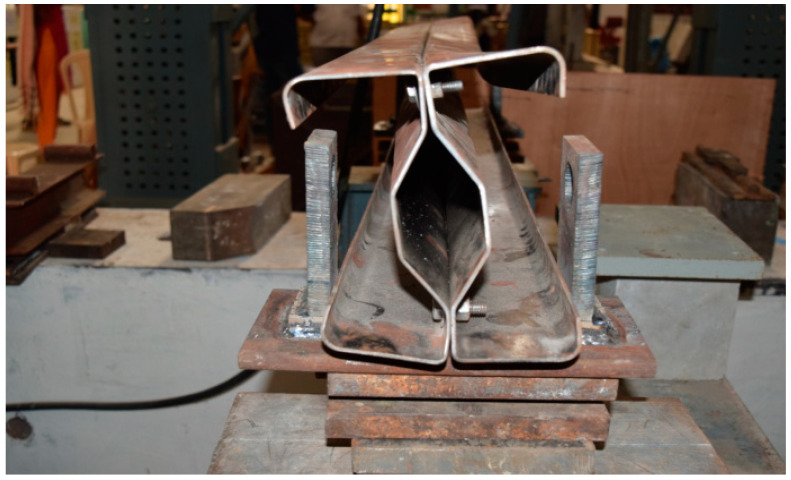
Failure mode B2B100.

**Figure 10:**
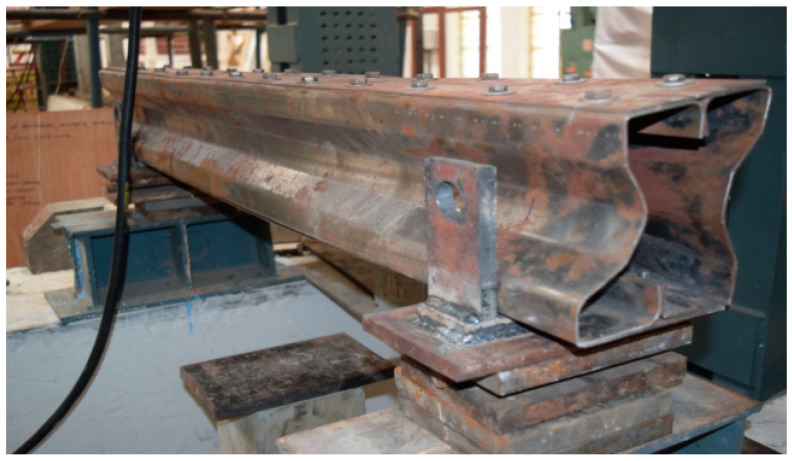
The failure mode of F2F100.

**Figure 11:**
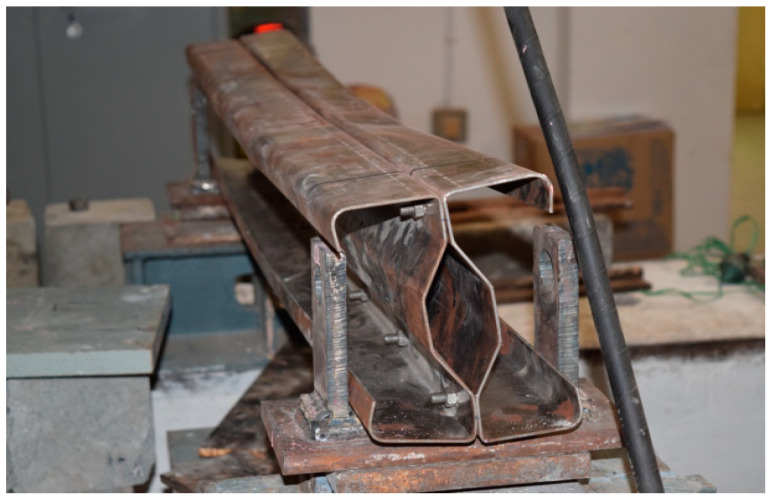
The failure mode of B2B157.

**Figure 12:**
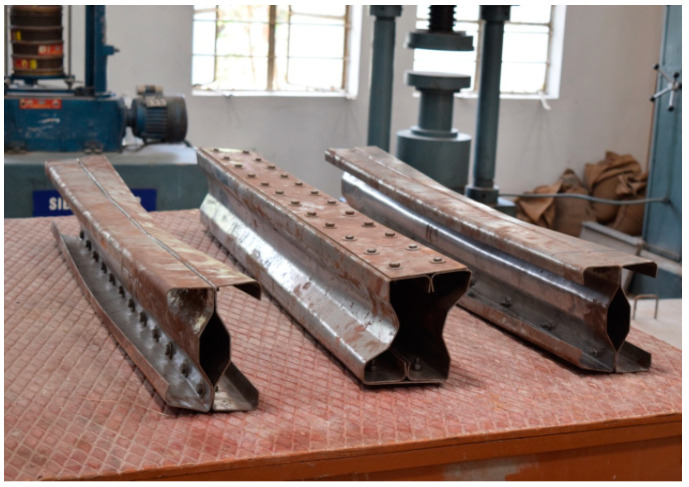
Tested specimens.

The specimen underwent local buckling and lateral-torsional buckling, as shown in the following figures. The buckling modes resemble the analytical results. Thus, the analytical results are verified experimentally. The failure modes of the tested specimens follow (Figure 9, Figure 10, Figure 11 and Figure 12).

### 3.3. Load–Deflection Behavior

**Figure 13:**
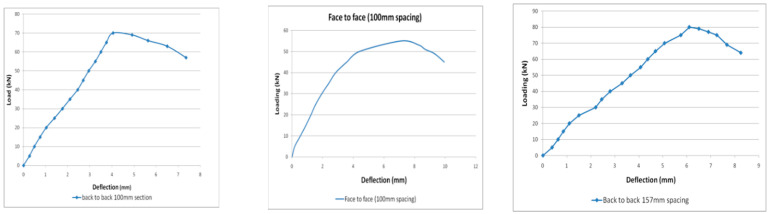
Load vs. deflection behavior.

The B2B100 section has an ultimate load of 70.21kN and the F2F100section has an ultimate load of 55.23kN, a 21.3% decrease in the load-carrying capacity compared with the B2B100 section. B2B157 is the modified back-to-back connected built-up section having a maximum load of 85.33 kN, which is a 21.6% increase in the load-carrying capacity compared with the standard B2B100 section (Figure 13).

### 3.4. Ultimate Load Carrying Capacity

**Figure 14:**
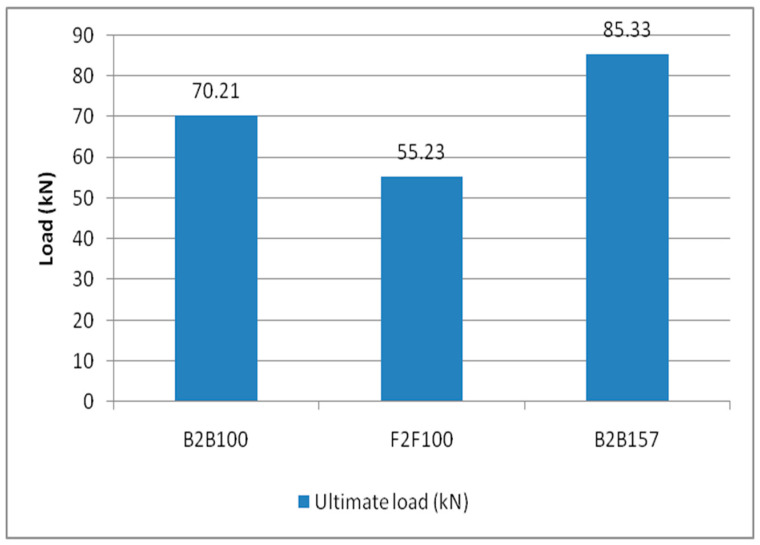
Ultimate load carrying capacity.

From the table, it is found that the B2B100 standard section has a critical load of 70 kN. The most effective section is B2B157, having a critical load of 85 kN, a 21.6% increase in load carrying capacity compared with the standard section B2B100. The local buckling and lateral-torsional buckling are the failure modes of the proposed cold-formed steel built-up sections. Thus, the analytical results are verified experimentally. Section B2B157 has the highest load carrying capacity among the three sections, and the load-carrying capacity is 21.6% higher than the standard B2B100 section (Table 2 and Figure 14).

## 4. Results and Discussions

### 4.1. Comparison of Ultimate Load-Carrying Capacity

**Figure 15:**
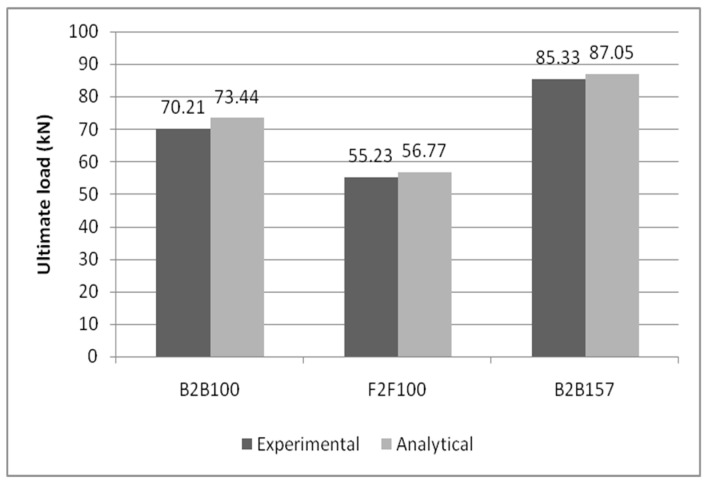
Comparative bar chart of the experimental and analytical results of ultimate load-carrying capacity.

The many criteria discussed included ultimate load bearing capability, buckling mode, and load–deflection behaviour. The loads corresponding to the maximum deflection for B2B100mm, F2F100mm, and B2B157mm were plotted for both analytical and experimental investigations. It is clear from Table 3 that the experimental method has a greater load value than the analytical method, especially in B2B157, which has 18.6% and 21.4% greater load carrying capacity than B2B100 in the analytical and experimental investigations, respectively, as shown in Figure 15. The results are comparable to the existing outcomes [14,15,16].

Thus, the load-carrying capability is improved by increasing the bolt spacing within the permissible limits, which eventually depicts similar findings to the literature sources [17,18,19].

### 4.2. Comparison of Load versus Deflection Curve

**Figure 16:**
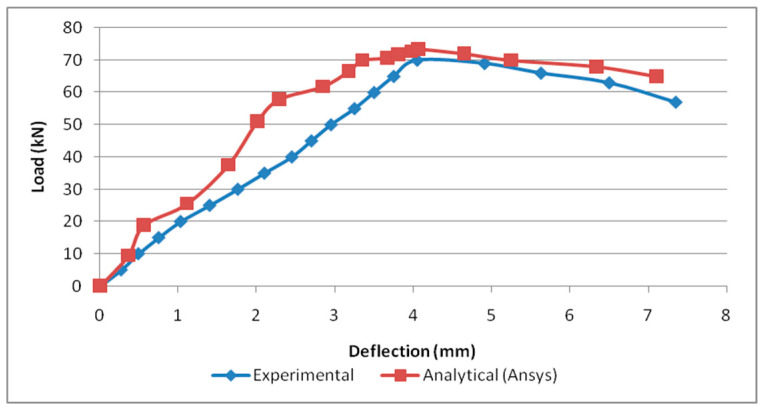
Load vs. deflection for B2B100.

**Figure 17:**
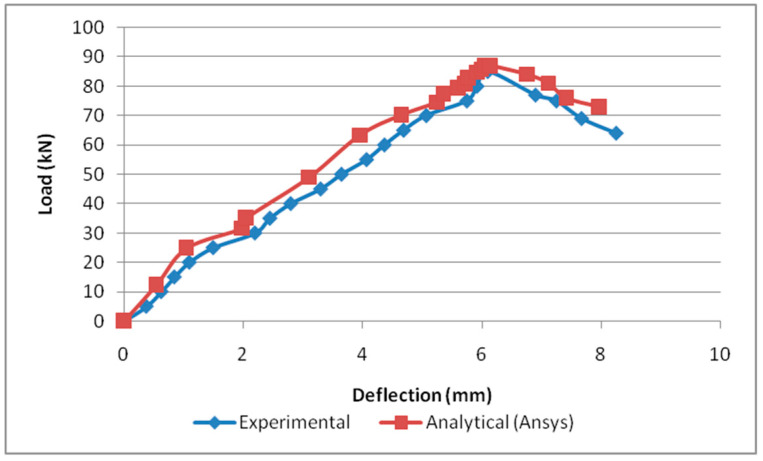
Load vs. deflection for B2B157.

**Figure 18:**
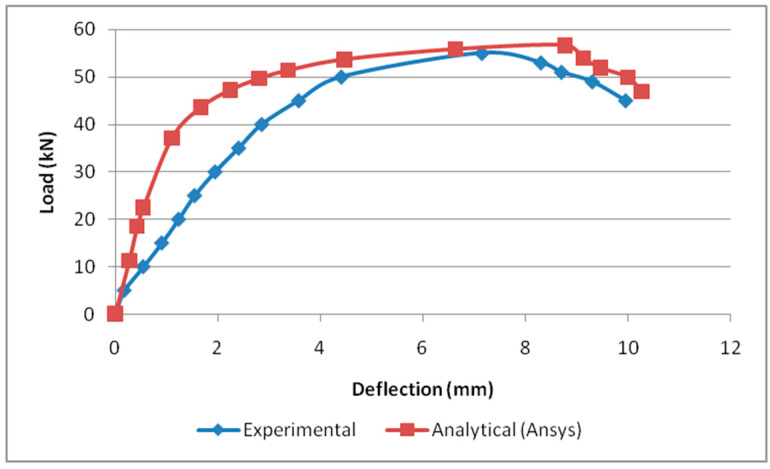
Load vs. deflection for F2F100.

The load correlating to the deflection comparison for specimens B2B100, B2B157, and F2F100 for both analytical and experimental behaviour was plotted as shown in Figure 16, Figure 17 and Figure 18. This analytical behavior has a somewhat greater value than the experimental behavior and, in addition, the outcomes exhibit a similar trend variation to the existing outcomes [20,21].

## 5. Conclusions

The analytical investigation focused on finding all three specimens’ ultimate load-carrying capacity. Finite element models were developed using ANSYS software 16.1 to study the buckling behavior of the fabricated sections. Specimen B2B157 was discovered to be the most effective section compared with the standard section B2B100. Thus, sections B2B100, F2F100, and B2B157 were fabricated, and a test was performed to find the ultimate load-carrying capacity and relation to the analytical results. This experiment study investigated in depth the behaviour of cold-formed steel built-up sections. To acquire a complete knowledge of the behaviour of the proposed cold formed steel built-up sections, both experimental and finite element analyses were used. The advanced finite element tool ANSYS was used to create finite element models of cold-formed steel built-up beams that were tested. The load–deflection curves and buckling modes from the test and the finite element analyses were compared to validate them. Cold-formed steel built-up beams were the subject of the experimental studies. The beams were put through their paces with a two-point load and a simply supported end condition. The failure modes predicted by finite element analysis match the failure modes observed in the tests very well.

- B2B157 is the most effective section among the proposed built-up beams, according to analytical and experimental data.
- The fabricated section B2B157 has a load-carrying capacity that is 21.6 percent higher than B2B100 for the same quantity of material.
- Load-carrying capability is improved by increasing the bolt spacing within permissible limits.
- The ANSYS software-based finite element model accurately predicts the strength and behavior of the beams. As a result, the finite element analysis may be utilized to predict the flexural member’s load capacity with a high confidence level. While designing cold-formed steel beams, local, distortional, bending, web buckling, and lateral-torsional buckling must be considered.
- The addition of a stiffened element at the web area and edge stiffeners at the flange significantly improves the flexural strength and behavior of the beams.

## Figures and Tables

**Table 1:** The ultimate load of built-up sections.

Specimen	Load Corresponding to Maximum Deflection (kN) P_Ansys_	% Variation in Strength	
		With Respect to B2B100	Considering C/S Area for F2F100
B2B100	73.44	-	-
F2F100	56.77	−22.6%	−52.5%
B2B157	87.05	18.6%	-

**Table 2:** The ultimate load of the built-up sections.

Specimen	Ultimate Load (kN) Experiment	% Variation in Strength	
		With Respect to B2B100	Considering C/S Area for F2F100
B2B100	70.21	-	-
F2F100	55.23	−21.3%	−51.7%
B2B157	85.33	21.6%	-

**Table 3:** The ultimate load-carrying capacity of specimens.

Specimen	Load Corresponding to Max Deflection (kN) P_ANSYS_	% Variation in Strength (Analytical)	Ultimate Load (kN) P_EXPERIMENT_	% Variation in Strength (Experimental)
B2B100	73.44	-	70.21	-
F2F100	56.77	−22.6%	55.23	−21.3%
B2B157	87.05	18.6%	85.33	21.6%

## Data Availability

No data were used to support this study.
